# Successful surgical management of Ménétrier-like disease in a Devon Rex cat

**DOI:** 10.29374/2527-2179.bjvm005625

**Published:** 2025-10-01

**Authors:** Evelina Burbaitė, Vytautas Stankus, Cynthia de Vries, Brigita Bandzevičiūtė, Vytautas Sabūnas

**Affiliations:** 1 8 Drambliai Veterinary Hospital, Vilnius, Lithuania; 2 Dr. L. Kriaučeliūnas Small Animal Clinic, Faculty of Veterinary, Veterinary Academy, Lithuanian University of Health Sciences, Kaunas, Lithuania; 3 Pathology Department, Laboklin GmbH & Co.KG, Bad Kissingen, Germany; 4 Veterinary Department, Faculty of Agrotechnology, Vilnius College, Vilnius, Lithuania

**Keywords:** cat, Ménétrier-like disease, partial gastrectomy, hypertrophic gastritis, gato, doença semelhante à de Ménétrier, gastrectomia parcial, gastrite hipertrófica

## Abstract

Ménétrier’s disease is a rare human condition that causes diffuse hypertrophy of the gastric rugal folds. A similar condition in dogs has been reported as Ménétrier-like disease (MLD). To date, only two feline cases have been reported, and treatment was either unsuccessful or not documented. A 3-year-old female spayed Devon Rex cat was presented with acute nausea, retching, and vomiting. Within two months, multiple tests were performed to reach the diagnosis. Macroscopically, during gastroduodenoscopy, cerebriform hypertrophic gastric mucosal folds were observed in the gastric fundus, cardia, and greater curvature of the stomach. Superficial biopsy sampling of the gastric mucosa showed mild mucosal oedema and low numbers of *Helicobacter* spp. Considering the course of the disease, persistent gastrointestinal symptoms, and quality of life, a partial gastrectomy was performed. Histology of full-thickness biopsies confirmed hyperplastic fibrosing gastropathy with multifocal cystic dilated gastric glands resembling Ménétrier-like disease in dogs. At the time of the 3-month follow-up, the cat was asymptomatic, and the gastroduodenoscopy was unremarkable. This case report presents clinical findings, challenging diagnostics, and a novel treatment option in a cat with hyperplastic fibrosing gastropathy. To the authors‘knowledge, this is the first reported successful surgical management of MLD in a cat.

## Resumo

A doença de Ménétrier é uma condição rara em humanos que causa hipertrofia difusa das pregas rugosas gástricas. Uma condição semelhante em cães foi descrita como doença semelhante à de Ménétrier (MLD). Até o momento, apenas dois casos felinos foram relatados, e o tratamento foi ineficaz ou não documentado. Uma gata Devon Rex de 3 anos, castrada, foi apresentada com náuseas agudas, engasgos e vômitos. No período de dois meses, diversos exames foram realizados para chegar ao diagnóstico. Macroscopicamente, durante a gastroduodenoscopia, observaram-se pregas mucosas gástricas hipertróficas cerebriformes no fundo, cárdia e curvatura maior do estômago. A biópsia superficial da mucosa gástrica mostrou edema mucoso leve e baixos números de *Helicobacter* spp. Considerando a evolução da doença, os sintomas gastrointestinais persistentes e a qualidade de vida, foi realizada uma gastrectomia parcial. A histologia das biópsias de espessura total confirmou uma gastropatia fibrosante hiperplásica com glândulas gástricas císticas dilatadas multifocais, semelhante à doença semelhante à de Ménétrier em cães. No acompanhamento de 3 meses, a gata estava assintomática, e a gastroduodenoscopia não apresentou alterações. Este relato de caso apresenta achados clínicos, desafios diagnósticos e uma nova opção de tratamento em um gato com gastropatia fibrosante hiperplásica. Até onde se tem conhecimento, este é o primeiro relato de sucesso no tratamento cirúrgico de MLD em um gato.

## Introduction

Ménétrier’s disease (MD) is a rare idiopathic human disease that causes hypertrophy of gastric mucosal folds, usually associated with hypoalbuminemia and hypochloremia (Coffey et al., 2007; Friedman et al., 2009; Scott Junior et al., 1975; Toubia & Schubert, 2008). Since its first description in 1888, multiple studies have been conducted, associating the condition with neoplastic disease and *Helicobacter* spp. infection (Badov et al., 1998; Bayerdörffer et al., 1994; Menetrier, 1888; Munday et al., 2012). In veterinary scientific literature, a similar condition has been described in dogs and named Ménétrier-like disease (MLD) (Lagerstedt et al., 2021; Lecoindre et al., 2012; Rallis et al., 2007; Romanucci et al., 2021; Sarandy et al., 2021). Most animals are presented at an adult or older age. The youngest reported MLD-affected dog was 4 years old (Vaughn et al., 2014), while other case reports state that the dogs were geriatric and 7 years old or older (Jang et al., 2021; Lagerstedt et al., 2021; Lecoindre et al., 2012; Munday et al., 2012; Rallis et al., 2007; Romanucci et al., 2021; Sarandy et al., 2021; van der Gaag et al., 1976). Most documented breeds were Cairn Terriers and Labrador Retrievers (Munday et al., 2012; Romanucci et al., 2021; Toubia & Schubert, 2008). No breed predisposition has been noted in cats so far. However, it is speculated that genetic predisposition is possible, as in two studies, affected animals were related (Munday et al., 2012; van der Gaag, 1984). It has also been reported that MLD might be associated with neoplastic diseases, particularly adenocarcinoma and sarcoma (Lecoindre et al., 2012; Munday et al., 2012; Romanucci et al., 2021; Sarandy et al., 2021), while MD in humans is related to Helicobacter infection (Badov et al., 1998; Bayerdörffer et al., 1994).

Dogs diagnosed with MLD exhibit gastric mucosa thickening of variable degree; therefore, the most commonly presented symptoms were vomiting, weight loss, anorexia, abdominal pain, and lethargy (Jang et al., 2021; Lagerstedt et al., 2021; Lecoindre et al., 2012; Munday et al., 2012; Rallis et al., 2007; Romanucci et al., 2021; Sarandy et al., 2021; van der Gaag et al., 1976; Vaughn et al., 2014). Upon examination, the typical laboratory findings were mild anemia, hypoproteinemia, and hypoalbuminemia (Lagerstedt et al., 2021; Lecoindre et al., 2012; Rallis et al., 2007; Romanucci et al., 2021; van der Gaag et al., 1976; Vaughn et al., 2014). Diagnostic imaging, such as abdominal radiography, ultrasonography, video-gastroscopy, and even computed tomography, is usually suggestive but undiagnostic. The final diagnosis is usually reached after performing a full-thickness biopsy histopathological examination, which displays mucous gland hyperplasia, parietal and chief cell atrophy, gastric gland cystic dilation, overall edematous, hypertrophic gastric mucosa (Coffey et al., 2007; Lagerstedt et al., 2021; Toubia & Schubert, 2008). Emerson et al. (2014) have described MLD in two red-capped mangabeys, who presented similarly to dogs, suffering from vomiting, diarrhea, and abdominal pain. The primates had similar laboratory findings- anemia, hypoproteinemia, and hypoalbuminemia, however, the identification of the pathology was very challenging and resulted in unsuccessful both medical and surgical treatment (Emerson et al., 2014). To the authors‘knowledge, up to date, only two cases of feline hyperplastic gastropathy presumed to be MLD have been reported (Barker et al., 2019; Dennis et al., 1987). The case reports were written more than three decades apart, yet both cats suffered an unsuccessful outcome. It is possible that the lack of medical reports and poor description leads to MLD being underreported and underrecognized. The purpose of this case report, therefore, was to describe the clinical and diagnostic imaging findings, as well as the surgical treatment procedure in a cat with MLD.

## Case description

A 3-year-old female spayed Devon Rex cat was presented for acute retching, nausea, and vomiting. General clinical examination, routine hematology, and radiographic study were unremarkable (Table 1).

**Table 1:** Laboratory examination findings during different time frames, presenting the most common hematology findings in affected animals.

	RBC (Red Blood Cell) Count	HCT (Haematocrit)	Alb (Albumin)	TP (Total protein)
At presentation	10.66 x 10^12^/L	0.446 L/L	36 g/L	89 g/L
At 2^nd^ gastroscopy	10.05 x 10^12^/L	0.423 L/L	37 g/L	83 g/L
Before surgery	9.50 x 10^12^/L	0.397 L/L	38 g/L	83 g/L
2 months after surgery	10.62 x 10^12^/L	0.449 L/L	34 g/L	81 g/L

Despite that, clinical symptoms were persistent and gradually worsening.

Eleven days after the initial presentation, the cat was scheduled for gastroduodenoscopy. Vital parameters, abdominal palpation, complete blood cell count (CBC), and serum biochemistry profile parameters were within range. The cat was sedated using 5 mcg/kg of dexmedetomidine (Dexdomitor 0.5 mg/mL, Orion Corporation, Finland) and 0.2 mg/kg of butorphanol (Butomidor 10 mg/ml, Richter Pharma AG, Austria) intravenously. Anesthesia was induced using 4 mg/kg of propofol (Propoven 10 mg/ml, Fresenius Kabi AB, Sweden) intravenously. Following the loss of the gag reflex, the animal was intubated using a 3.5 mm endotracheal tube, which was then cuffed. Gastroscopy was executed using an Olympus GIF-H180 gastroduodenoscope (Olympus Corporation, Japan). Upon passing the esophageal sphincter, the stomach showed marked rugal fold thickening at the level of the gastric fundus, cardia, and greater curvature. Using endoscopic biopsy forceps, gastric and duodenal mucosal samples were taken for in-house cytological examination.

Smears showed good preservation, high cellularity, in an eosinophilic background. Columnar epithelial cells of normal size, with single round nuclei showing isocytosis and isokaryosis, were present. Mitoses could not be observed. A round cell population composed of approximately 70% plasma cells, 15% non-degenerate neutrophils, 10% eosinophils, and 5% small lymphocytes. Fungal hyphae and protozoa were absent. High numbers of extracellular organisms resembling *Helicobacter* spp. were seen. Abundant extracellular hematoidin crystals were visible.

Based on these findings, a chronic active lymphoplasmacytic to mixed gastritis with concurrent *Helicobacter* spp. presence was suspected. Supportive therapy with proton‐pump inhibitors, amoxicillin-clavulanic acid, and prednisolone (0.7 mg/kg BID, tapered weekly) was initiated.

**Figure 1:**
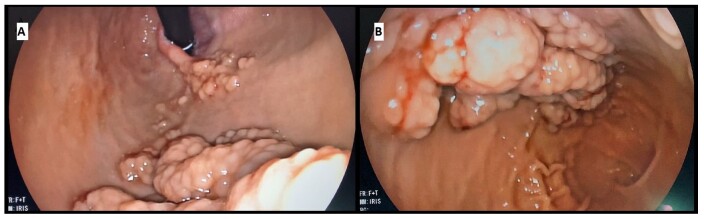
Images of the second gastroscopy in a 3-year-old female spayed Devon Rex cat displaying the abnormal gastric wall. The gastric lesions extend along the gastric fundus, cardia (A), and most of the greater curvature (B).

As a response to the treatment was lacking, gastroduodenoscopy was repeated after 1 month. Endoscopically, markedly enlarged gastric folds were visible in the gastric fundus, cardia, and most of the greater curvature. The rugal folds had a cerebriform-like appearance (Figure 1). Superficial gastric mucosal biopsy samples were repeatedly taken and submitted to Laboklin GmbH & Co.KG. Non-specific mild multifocal superficial oedema of the gastric mucosa was reported. A low to moderate number of spiral-shaped organisms compatible with *Helicobacter* spp. were observed.

After extensive scientific literature analysis, MLD was suspected, therefore, the surgical treatment was proposed and accepted by the owners. Before the surgery, a full-body computed tomography (CT) scan was performed, which was unremarkable and displayed no changes in internal organs or regional lymph nodes. Considering the growth of the lesion and the worsening of the clinical course, a third gastroscopy was performed as well, which displayed a macroscopically identical appearance of the gastric mucosa.

A total of 53 days after the initial presentation, the animal was admitted for a partial gastrectomy. The cat was sedated using 5 mcg/kg of dexmedetomidine (Dexdomitor 0.5mg/mL, Orion Corporation, Finland) and 0.2 mg/kg of methadone (Insistor 10 mg/mL, Richter Pharma AG, Austria) intramuscularly. Anesthesia was induced using 4 mg/kg of propofol (Propoven 10 mg/mL, Fresenius Kabi AB, Sweden) intravenously and furtherly maintained using sevoflurane 2% in 2L of pure oxygen. After trichotomy and aseptic surgical field preparation, a cranial celiotomy was performed. Upon gastrotomy, an ill-defined cerebriform neoformation was seen on the gastric mucosa and removed with clear margins of 1 cm. It was compatible with the gastric changes observed on gastroduodenoscopy. The adjacent liver, intestines, diaphragm, and abdominal wall did not display any macroscopic changes. The removed gastric wall neoformation reached maximum dimensions of 64 × 25 × 8 millimeters in length, width, and thickness, respectively. Considering the irregular shape and form of the removed lesion, the gastric wall was stitched in a Y-shaped suture. Polydioxanone 3-0 suture material was used for mucosal and serosal gastric layers, 2-0 for the transverse fascia and abdominal wall, while poliglecaprone 25 was used for the subcutis and intradermal skin suture. Postoperatively, the cat was dressed in a postoperative protective garment, and low-fat gastrointestinal feed was prescribed in small amounts every 4 hours. Antibiotic therapy was prescribed for preventative measures (amoxicillin and clavulanic acid at 22mg/kg OS BID), and NSAIDs (meloxicam 0.1 mg/kg OS SID) were given for 3 days.

The gastric neoformation and mesenteric lymph node samples were fixed in 10% neutral-buffered formalin and routinely processed for histopathology at Laboklin GmbH & Co.KG.

**Figure 2:**
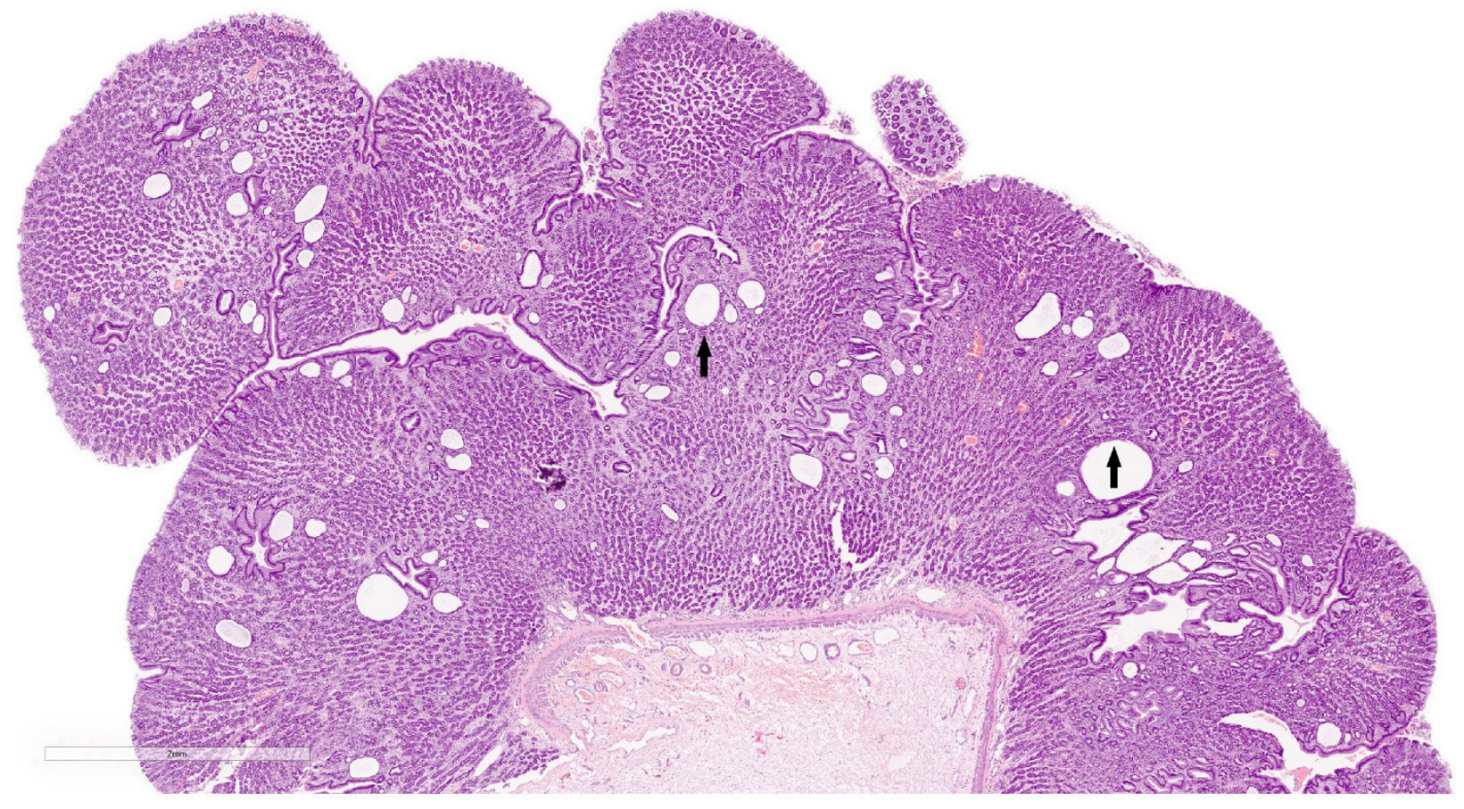
Overview of a diffuse marked thickening of the mucosal layer, with multifocal cystically dilated mucosal glands (arrows). HE, 2x magnification.

**Figure 3:**
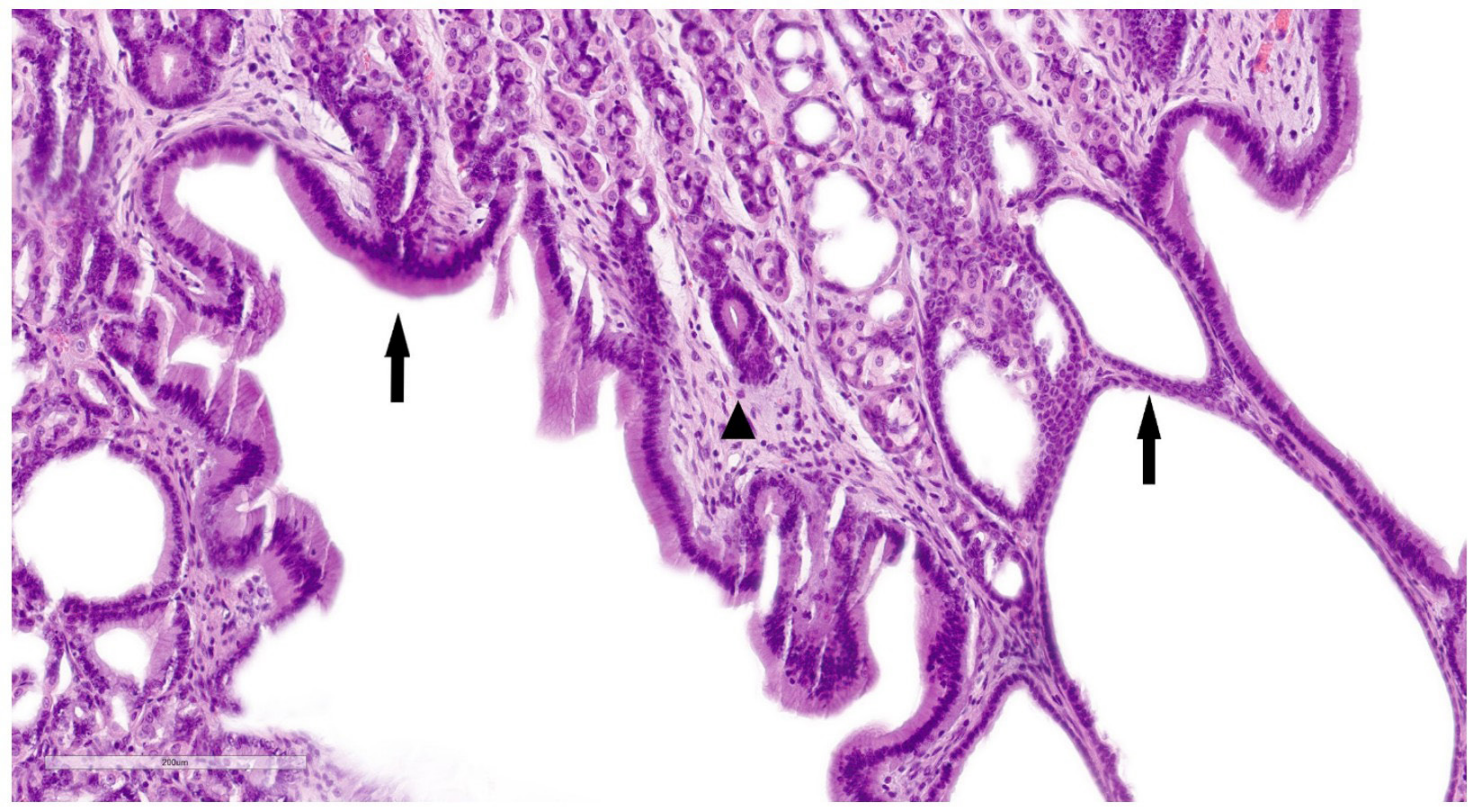
Cystic mucosal glands are lined by flattened to low cuboidal epithelial cells (arrows). There is a mild lymphoplasmacytic inflammatory infiltrate visible in the mucosal propria (triangle). HE, 20x magnification.

On histology, diffuse marked thickening of the mucosal layer was visible. The surface columnar epithelium was multifocally ulcerated but unremarkable in the intact regions. Throughout the mucosa, low numbers of variably sized, cystically dilated mucosal glands were lined by a single layer of flattened to cuboidal epithelial cells (Figures 2 and 3). In a few areas, there was mild chief cell hyperplasia with loss of parietal cells. The mucosal propria was multifocally mildly expanded by a low number of lymphocytes, fewer plasma cells, and rare neutrophils, with multifocal mild superficial mucosal fibrosis. The submucosa showed mild oedema and congestion. The tunica muscularis was unremarkable. The mesenteric lymph node showed mild lymphoid follicular hyperplasia. All findings were consistent with hyperplastic fibrosing gastropathy with cystic glandular ectasia, resembling canine Ménétrier-like disease.

Two months after the surgery, a control gastroduodenoscopy was performed. The mucous membrane was smooth and unremarkable. Scar tissue formation at the gastrectomy site was consistent with postoperative changes.

## Discussion

Ménétrier-like disease seems to be a veterinary medicine substitute for human Ménétrier’s disease. The pathology in both humans and dogs, cats, and primates displays similar characteristics in clinical symptoms, laboratory findings, and macroscopic gastric lesion appearance. Just as veterinary patients, humans can present with abdominal pain, vomiting, and diarrhea, and display hypoproteinemia together with hypoalbuminemia on laboratory testing (Friedman et al., 2009; Scott Junior et al., 1975). It has been speculated that protein and albumin loss in MD is a consequence of gastric tight junctional complex widening, and therefore, this protein-losing gastropathy is a potentially lethal disease (Kelly et al., 1982; Scott Junior et al., 1975). However, there are reports of successful surgical resections of gastric protein loss sites within the stomach wall in both humans and dogs (Jang et al., 2021; Lecoindre et al., 2012; Scott Junior et al., 1975; Vaughn et al., 2014). According to the authors' understanding, this is the first successful surgical MLD treatment in a feline patient.

Just as previously described, gastric protein-losing with consecutive hypoproteinemia, hypoalbuminemia, and anemia were the most expected laboratory findings in affected cats (Barker et al., 2019). The cat in this report, however, did not present any of these changes. A feline MLD study by Barker et al. (2019), however, has reported that the affected cat was symptomatic for 6 months before presentation and had suffered severe weight loss. In contrast, the current patient was brought in on the day of symptom presentation by attentive owners who arrived during the night shift of the veterinary emergency service. It is possible that attentive animal caregivers and owners are able to recognize early symptoms of illness and seek medical assistance promptly, preventing the progression of the disease. Early intervention might be the reason why, in the present case, concurrent hematological changes were not present.

Limited literature resources and the rarity of the condition might cause significant challenges in clinical practice. The current case report displays that multiple diagnostic imaging studies and tissue samples were needed to reach the diagnosis, and these findings agree with the previous studies (Coffey et al., 2007; Emerson et al., 2014; Lagerstedt et al., 2021; Toubia & Schubert, 2008). Both Lagerstedt et al. (2021) and Emerson et al. (2014) have reported that 3 and 5 gastroduodenoscopies, respectively, were needed to better characterize the gastric lesions. It is also worth noting that histological examination of a full-thickness gastric wall biopsy is necessary for a final diagnosis since endoscopic biopsy instruments are too superficial to accurately represent the affected tissue (Barker et al., 2019; Coffey et al., 2007; Lagerstedt et al., 2021; Toubia & Schubert, 2008). This is the reason why our initial attempts at tissue sampling with endoscopic forceps were not fruitful, but full full-thickness biopsy was conclusive.

Research suggests an association between the presence of Helicobacter-like organisms and Ménétrier’s disease in humans or MLD in dogs. To our knowledge, this study describes the first concurrent helicobacteriosis with feline MLD; however, definitive histological and immunochemistry examinations were not performed. After medical treatment for *Helicobacter* spp. infection, the cat did not show clinical improvement, which is comparable to the canine study (Lagerstedt et al., 2021). Considering the high incidence of *Helicobacter* spp. isolation in asymptomatic cats, it remains possible that the species is a part of the physiological feline gastric microbiome (Taillieu et al., 2022).

Although not yet described in cats, some canine and human cases of Ménétrier’s disease were suspected to be an initial presentation of gastric tumors such as sarcoma (Romanucci et al., 2021), carcinoma (Lecoindre et al., 2012), and adenocarcinoma (Munday et al., 2012; Sarandy et al., 2021). The potential premalignant nature of gastric lesions was part of the reason to opt for a surgical removal of the affected gastric mucosa. Even if the current findings did not show any signs of malignancy, thorough clinical follow-up of the patient is advised to identify possible remissions.

We acknowledge that the current study has several limitations. First, helicobacter-like microorganisms were only identified morphologically; molecular confirmation was not performed. Secondly, immunohistochemistry malignancy testing of the excised stomach wall was not performed. And lastly, diseases that are chronic in nature usually require a long-term outcome to prove their effectiveness. In our case, the last follow-up was performed two and a half months after the surgery.

## Conclusions

This case report presents a rare case of suspected Ménétrier-like disease in a cat, its clinical characteristics, a difficult diagnostic journey, and a new surgical treatment option. Considering the challenging diagnostic workup of suspected Ménétrier-like disease, we do suggest performing a full-thickness gastric wall histological examination for the final diagnosis, as superficial biopsy can rarely assist in the diagnostic workup. We do conclude that partial gastrectomy seems to be a viable and novel treatment option in cats diagnosed with Ménétrier-like disease. However, prompt action and early intervention are advised to minimize the secondary effects of the disease itself. Finally, additional studies with longer follow-up intervals should be performed to evaluate the effectiveness of partial gastrectomy in cases of feline Ménétrier-like disease.
